# Thoughts on Fang Filling

**Published:** 1859-11

**Authors:** Geo. Watt


### THOUGHTS ON FANG FILLING.

BY GEO. WATT.

IN the July number of the "Register" we made a few remarks on the subject now under consideration, which have already given rise to some discussion. The article is, perhaps, carelessly worded, or, if not, it has been carelessly read in some cases. The former view is likely the correct one, as we write such with more haste than we do our regular chemical articles.

Some have inferred that we maintain that the orifice in the apex of the fang will be uniformly closed with osseous matter. Our remark does not warrant the conclusion. We simply said, "As to the foramen in the end of the fang, nature closes it up better than can be done with gold." But this is true whether it be closed with osseous or fibrous tissue.

Dr. Broch, in the *American Dental Review*, in speaking of our method, says he "inserts a little cotton, with creosote, in the nerve cavity, and plugs over this." Dr. B. should remember, that this is Dr. Atkinson's "thunder," approved, however, by us at the Niagara meeting, according to all the "reports" we have yet seen, except the "*Register's*," because the creosote forms a "carbonate," by uniting with the cotton!

Do the editors of the various journals think that I regard *carbonic* acid as the active principle of creosote? or shall I conclude that they don't know that pure creosote is *carbolic* acid? or shall we blame it all on the poor printers?

But Dr. B. suggests that the practice is not venerable, and Dr. McQuillen, in the "*Cosmos*," hints at the same thing. And as we reported recent cases, only there is a shadow of a foundation for their inference. The object aimed at was to report only such cases as we could see or hear from frequently, and keep the profession posted up, from time to time, on these. We are again ready to report on them, having seen all but No. 9, the present week. Will the reader turn to the first article of the July number and compare?

CASE 1.—As it was—neither pain nor abscess.

CASE 2.—All right.

CASE 3.— Do.

CASE 4.—No uneasiness.

CASE 5.—Slight periostitis last week in one—the other still easy and all right.

CASE 6.—Has never been uncomfortable.

CASE 7.— Do. do. do.

CASE 8.—As last reported.

CASE 9.—Patient died in August—the tooth right till then.

We will report no new cases at present; but will remark in this connection, that the mode of practice under consideration is not *very* recent with us. At a meeting of the Mississippi Valley Association, in 1857, perhaps, we mentioned it as our favorite method, and it was not new then.

But Dr. B; if we understand him, agrees to leave some dead matter in the canal. The "extreme end" is not a part of his creed. He says, "The leading law of success, I believe, is the complete removal of pulp and lining membrane as near as possible to this extreme point. Do this, and you need not make the tooth a charcoal factory, or a tan pit. It will do well, even if you can not fill it to the point extreme."

Every operator will admit that in some cases the "extreme end" can be readily reached. In these, prolonged treatment is not called for. But the practice we described in the July number is based on the assumption that it is not practicable to thoroughly cleanse the entire canal. And this treatment does not make "a charcoal factory" of the canal, and, if it did, it would be no objection to it, as charcoal in the cavity could do no harm.

But after all, what is the real difference between Dr. B.'s treatment and ours? He fills a part of the canal; we fill none of it. He fills over putrescent matter, if he can't remove it; we arrest and prevent its putrefaction. He cleanses out the canal as well as he can, and we do—the same.

Dr. B. does me injustice in stating that, according to my article, fang filling became fashionable in 1853. I merely say it "has been fashionable since, and probably was before," and state in evidence of the latter idea, that "a majority, if not all the members present, advocated filling the fangs."

In conclusion, now, we would state that, though in speaking of the treatment under consideration, we have called it "our mode," we do not wish it to be understood that we claim it as peculiar to us. We are glad that others have investigated the subject, and arrived at the same conclusions.

OCTOBER 1, 1859.

